# Prevalence of alcohol and substance use relapse in forensic inpatients with substance use disorders in Germany

**DOI:** 10.3389/fpsyt.2025.1663413

**Published:** 2025-10-17

**Authors:** Seyma Düger, Finn Sörensen, Birgit Völlm

**Affiliations:** ^1^ LVR-Clinic, Department for Forensic Psychiatry V, Bedburg-Hau, Germany; ^2^ Clinic for Forensic Psychiatry, Rostock University Medical Center, Rostock, Germany

**Keywords:** substance use, forensic psychiatry, relapse, recurrence of substance use, predictors of substance use

## Abstract

**Background:**

Offenders who pose a risk of harm and whose convictions are linked to substance use can be mandated to undergo treatment in forensic psychiatric hospitals under Section 64 StGB of the German Penal Code (Strafsgesetzbuch; StGB), if there are reasonable prospects that treatment might be successful. Relapses during treatment is a common occurrence in patients with substance use disorders but little is known about the frequencies of such events in a forensic setting.

**Purpose:**

This study aimed to determine the prevalence of relapse among patients who undergo treatment under § 64 StGB, identify substances involved and possible predictive factors.

**Method:**

We utilized data over the span of two years from 108 patients who were admitted to the Clinic of Forensic Psychiatry in Rostock, Germany, between 2019 and 2021.We used descriptive statistics and multiple regression analysis. A relapse was defined as a positive laboratory test for illicit drugs or alcohol, admission of relapsing or a declined test (i. e. the patient did not consent to the test).

**Results:**

We found that 65.7% of the patients relapsed within the initial two-year period of stay. Cannabinoids were the most commonly consumed substances. Factors such as age, education level, comorbidity, number of previous convictions, duration of stay and type of substance used did not significantly affect relapse rates. Results are limited by a small sample size.

**Conclusion:**

A high relapse rate is still a reality of forensic addiction treatment. Static factors alone might only have a small predictive value for substance relapses and are not sufficient to fully predict individual risk. Therefore our findings show a need to focus on dynamic factors that affect consumption relapse rates. Considering the findings of this research, future studies should investigate dynamic factors of the patient’s substance use behavior during treatment as a whole (e.g. reason for relapsing, choice of drug etc.), identify and investigate other factors affecting relapse rate and uncover possible treatment interventions that might reduce relapse rates, dropout rates and criminal recidivism.

## 1 Introduction

### 1.1 Mandatory treatment in forensic psychiatric hospitals in Germany

In Germany, people who commit criminal offenses due to a substance use disorder (SUD) or an alcohol use disorder (AUD) can be mandatorily admitted to a forensic psychiatric hospital under Section 64 of the German Penal Code (Strafgesetzbuch; StGB). In addition to a causal relationship between the disorder and the criminal offense, the main prerequisites for mandatory treatment are a sufficient chance of success and a high risk of reoffending if the disorder is not treated (1). The ultimate aim of mandatory treatment under § 64 StGB therefore is to reduce the risk of reoffending, which is achieved by treating the disorder and target criminogenic needs (2). Placement in a forensic mental health service, where treatment is delivered under § 64 StGB, is becoming increasingly common. Between 1995 and 2020, the number of patients who underwent mandatory treatment under § 64 StGB rose from 1,373 to 5,280 (3). The duration of treatment increased by an average of six months between 1995 and 2016, so that an average treatment duration of around 23 months was recorded for 2020 (4). However, approximately half of the patients do not successfully complete treatment (5) and evidence for the effectiveness of mandatory treatment under § 64 StGB is mixed. On the basis of increasing the number of patients Section 64 was reformed in June 2023. Mandatory treatment should now be limited to patients with a severe substance use disorder and a clear indication of likely treatment success (6).

### 1.2 Recurrence of substance use during forensic psychiatric treatment

In their systematic review Tomlin et al. (7) investigated eleven studies which reported on recurrence of substance use during forensic treatment and concluded that on average approximately half of the patients relapsed with a range from 16.7 to 81.6%.

Single studies contributing to the review include, e. g.: Schalast (8) who highlighted that 80% of patients undergoing treatment in a forensic psychiatric hospital in Germany for alcohol dependency experienced at least one relapse with 31% relapsing more than five times. Seifert and Leygraf (9) documented a 30% relapse rate across multiple forensic hospitals with the following relapse frequencies: 6.9% had a single relapse, and 16.7% relapsed two to four times. Schalast (10) observed that 55% of forensic patients relapsed during their treatment, with a higher rate of relapse among SUD compared to AUD patients within the initial twelve months of treatment. Körkel and Schindler (11) found that roughly 33% of patients relapsed during treatment. Berger (12) found a relapse rate of 16.7% in forensic patients. Knecht and Claßen (13) reported that it has not been possible to reduce the relapse rate below 20% during mandatory treatment at a forensic psychiatric hospital in Hamburg, Germany. Hartl (14) reported a relapse rate of 54.5% forensic psychiatric clinic in Regensburg, Germany. 14% of patients experienced one relapse, 12% two, 20% three to six, and 8% had six or more relapses. In their systematic review Tomlin et al. (7) further reported that across nine studies roughly 60% of forensic patients relapse after discharge within a 12 to 48 months follow-up. The latest study on recurrence of substance use reported a relapse rate of 50% within the first 32 months after discharge with substance use during treatment, personality disorder (PD) and previous convictions as their predictive factors (15).

### 1.3 Recurrence of substance use as a dynamic risk factor

These findings indicate that recurrence of substance use has not yet been successfully addressed in treatment. Furthermore, recurrence of substance use during therapy has different effects on treatment. Less or no relapses, high treatment motivation and treatment goal pursuit have been shown to predict good treatment outcome and lower termination rates in forensic settings (16–18). Relapses can either extend the duration of mandatory treatment (19) or can often lead to its early termination (20), which increases the likelihood of reoffending (21). It should be noted that relapses during forensic treatment do not automatically lead to a higher risk of reoffending and it has been shown that the risk of reoffending of patients who remain abstinent and those who relapse during therapy is similar in the long term (22). Undertakings to identify predictors of recidivism yielded mixed evidence at best and dynamic risk factors for recidivism have been insufficiently considered in favor of static predictors with regard to the therapeutic success of treatment (7). A better understanding of recurrence of substance use during treatment and its predictive factors therefore seems in order and could help to access to what extent relapses jeopardize the ultimate therapy goal of reducing the risk of reoffending (23).

### 1.4 Predictors for recurrence of substance use during treatment

Evidence of factors that predict recurrence of substance use during treatment is sparse. In non-forensic settings polysubstance users, e. g. individuals who use multiple substances, generally experience higher relapse rates, have shorter abstinence periods and are twice as likely to terminate treatment early compared monosubstance users, who only consume one type of substance (24–26).

In forensic settings older patients generally are more likely to maintain abstinence during and after treatment and have shorter treatment durations (14, 19). Hartl (14) reported that education level as well as the number of previous convictions also seem to be correlated to recurrence of substance use. Patients with low-level secondary school qualifications tend to stay abstinent longer and relapse less often compared to those with special needs qualifications. Patients with five or less previous convictions not only are more likely to stay abstinent but are also more likely to successfully complete treatment (70%) compared to patients with at least six previous convictions (64%). The number of previous convictions or a comorbid PD also play a role in recurrence of substance use after forensic treatment (15).

### 1.5 Objectives, aims, and research questions

Since the most recent study on this subject is a decade old, the primary purpose of this study is to provide up to date information on the prevalence of substance use relapse in § 64 StGB inpatients in Germany. Based on the reported literature, it seems reasonable to assume that patients with a SUD, a comorbid PD, lower education level, a younger age and more than five previous convictions have a higher chance of relapsing. Since evidence on these factors is mixed and some are not even from a forensic setting, a second goal of this study is to verify the influence of these factors on recurrence of substance use during forensic treatment and combine them into risk profiles. In the future, the use of such risk profiles might help to determine how many patients have a higher risk of relapsing, adapt treatment according to patients individual needs and increase their chances of completing treatment successfully.

Primary objectives:

1. Determine the current prevalence of substance use relapse among patients detained under § 64 StGB in one forensic hospital in Germany.
2. Identify the types of substances used.
3. Investigate if there is a difference in relapse between polysubstance users and monosubstance users.
4. Investigate if factors such as age, education level (no school qualification, secondary school qualification or school for children with special needs), alcohol use or drug use disorder, a comorbid personality disorder, number of previous convictions, duration of treatment and type of substance user (polysubstance versus monosubstance users) affect relapse rates during treatment and then combine them into a risk profile. Based on the literature above we derived the following hypotheses:

1. Relapse rates will decrease with age.
2. Relapse rates will increase as education levels decrease, with lower-qualified patients relapsing more frequently.
3. Polysubstance use is positively associated with higher relapse rates compared to monosubstance use.
4. Higher numbers of previous convictions will increase relapse rates.
5. SUD is associated with higher relapse rates compared to AUD.
6. A comorbid personality disorder is positively associated with relapse rates.
7. Relapse rates will decrease with duration of stay.

## 2 Methods

### 2.1 Design, setting and participants

This study was conducted at the Clinic for Forensic Psychiatry (KFP) located in Rostock, Germany. The KFP in Rostock accommodates 103 in-patients. The clinic has 7 wards overall. Patients are treated on an inpatient basis for a period of approximately two years in closed wards. When discharged on probation, patients continue to receive treatment in an outpatient setting. The vast majority of the patients have committed offenses in connection with a substance use disorder and are accommodated in accordance with § 64 StGB. Patients who were admitted under § 64 StGB between 2019 and 2021 were included in this study.

### 2.2 Data collection

Data was collected via the clinics electronic recoding system, where information regarding the patients’ ongoing treatment, clinical information and personal history as well as relapses is entered. The general patient information and entries concerning relapses were anonymized and transferred to an excel spreadsheet which was then used for further analysis.

### 2.3 Substance use relapse

In our study, we only considered relapses that occur during inpatient treatment. The reason for this is that relapse during treatment is a risk factor for discontinuation of treatment (20) and recidivism (27). We wanted to investigate how many patients are affected by this and what increases the likelihood of relapse during treatment.

A relapse was considered as a positive test result from a laboratory or if patients declined a drug test and did not admit drug use or if they admitted that they had consumed drugs. Laboratory tests included saliva and/or urine tests. To check for alcohol consumption, the results from the alcohol breath test and ethylglucuronide (EtG) values were looked at. Depending on the drug and the time during which the consumption is still detectable by a test, two positive results within a period shorter than three days between them were either considered as multiple relapses (for stimulants and opioids) or a singular one (for cannabinoids and alcohol with decreasing test scores).

### 2.4 Data analysis

For the first two research objectives (determine prevalence of relapse and identify types of substances used) descriptive analysis was used and the results are presented using frequencies and percentages. For the last research objective (effect of predictive factors on substance use relapse), we used multiple linear regression analysis with substance use relapse as the dependent (outcome) variable. Type-I error was set at 5%, type-II at 20%, which is generally recommended (28). It seemed reasonable to identify and include factors in the risk profile that have a medium effect on relapsing at least. *A priori* power analysis revealed that a sample size of 118 is required to at least detect an effect of medium size. Duration of stay, age, school qualification, diagnosis, number of Previous convictions, and the type of substance use were the independent variables (predictors). Dummy coding was used to incorporate nominal variables (school qualification and main diagnosis) into the regression model. The regression analysis was performed using IBM SPSS Statistics version 29.0.

## 3 Results

### 3.1 Descriptive analysis

The Results are shown in **Table 1** and **Table 2**. Upon admission patients were on average 33.1 years old and had an average of 9.4 previous criminal convictions and an average duration of stay of 1.8 years. Most patients had a lower-level school qualification, had a substance use disorder, consumed multiple substances and had no comorbid PD.

**Table 1:** Categorical sample characteristics.

Variables	Codes	N	Percentage
School Qualification level*	1: None 2: special needs school qualification 3: lower primary school qualification 4: medium primary school qualification 5: higher primary school qualification	29 8 46 23 2	26.8 7.4 42.6 21.3 1.9
Total		101	100
Main substance use disorder	1: Substance use disorder 2: Alcohol use disorder 3: Combined disorder	85 20 3	78.7 18.5 2.8
Total		101	100
Comorbid PD Total	1: Yes 2: No	31 77 101	28.7 71.3 100
Type of substance use	1: Monosubstance use 2: Polysubstance user	30 78	27.8 72.2
Total		101	100

*Level 2: no school qualification, secondary school qualification or school for children with special needs, in Germany called “Förderschule”. Level 3 “Hauptschule” (lower qualification level), Level 4 “Realschule” (medium qualification level). Level 5: “Gymnasium” (high qualification level).

**Table 2:** Metric sample characteristics.

Variables	Mean	Range
Age in Years	33.1	16 – 56
Previous convictions	9.4	0 – 29
Duration of Stay in Years	1.8	0.5 – 4.3
Relapses	3.4	0 – 32

### 3.2 Relapse frequencies

The results are shown in **Table 3**. In total 71 out of 108 patients relapsed during the first two years of stay after admission, which equals an overall relapse rate of 65.7%. Of those 71 patients who relapsed, 29.6% relapsed only once, and 30.9% relapsed more than five times. Overall relapses ranged from 0 to 32 relapses over the course of treatment with roughly 4 relapses on average for every patient in the clinic. The most common substances used when relapsing were cannabinoids, specifically, THC (37.9%) and Spice (8.9%). Furthermore, in 4 cases patients solely refused to take a drug test. 19 patients either had positive drug test results, voluntarily admitted relapsing or refused a drug test at times. 48 patients either had positive drug test or voluntarily admitted relapsing and never refused a drug test. In 15 cases test results were positive for more than one substance.

**Table 3:** Frequencies of relapses and substances used.

	*N*	Percentage
Relapses		
Yes	71	65.7
No	37	34.3
Total	108	100
Relapse frequencies		
Relapsed once	21	29.6
Relapsed twice	15	21.1
Relapsed 3 times	6	8.5
Relapsed 4 times	7	9.9
Relapsed ‗ 5 times	22	30.9
Total	71	100
Substances identified when relapsing		
THC	30	37.9
Spice	7	8.9
Amphetamine	16	20.3
Alcohol	10	12.7
Cocaine	8	10.1
Opioids	8	10.1
	79	100
Drug test refusal		
Patients refused drug test (counted as a relapse)	4	5.6
Patients had positive drug test, voluntarily admitted relapsing and refused a drug test at times	19	26.8
Patients had positive drug test, voluntarily admitted relapsing and never refused a drug test	48	67.6
Total	71	100

Furthermore, for each participant, the total number of tests done in every year was counted. These tests only include laboratory tests (urine, saliva), excluding rapid tests. **Table 4** shows the total number of tests done between the years 2019 and 2021, as well as the positive tests in that year.

**Table 4:** Total number of tests done between 2019–2021 in § 64 forensic inpatients.

Years	Number of tests	Percentage of positive tests
2019 2020	141 371	12.7 11.6
2021	659	16.2

### 3.3 Regression analysis

Most of the assumptions were met. Descriptive analysis showed that homoscedasticity might be violated, so we used the HC3-Method in SPSS to control for heteroscedasticity. The regression analysis yielded a negative result, with F (10, 97) = 1.030, p‗.05 (**Table 5**) and a negligibly small effect with an adjusted R^2^ = 0.003 (**Table 6**) that, if significant, could explain a mere 0.3% of the variance. None of the investigated predictors affected consumption relapse rate, with p>.05 (**Table 7**).

**Table 5:** Independent sample test.

ANOVA^a^						
Model		Sum of squares	df	Mean square	F	Sig.
1	Regression	262.093	10	26.209	1.030	.424^b^
	Residual	2467.574	97	25.439		
	Total	2729.667	107			

^a^Dependant Variable: Total_relapses.

^b^Predictors: (Constant), Duration, BZR_entries, SUD, Hauptschule, Comorbid_PD, Förderschule, Type_of_substance_user, Age, Realschule, AUD.

**Table 6:** Model summary.

Model	R	R square	Adjusted R square	Standard error of the estimate	Durbin-watson-statistic
1	.310a	.096	.003	5.044	1.983

a. Dependant Variable: Total_relapses.

**Table 7:** Parameter estimates with robust standard errors.

Dependent variable: Total_relapses							
Parameter	Regression coefficient (B)	Robuster standard error^a^	t	Sig.	95% Confidence interval		Partial eta squared
					Lower bound	Upper bound	
Constant	6.664	5.242	1.271	.207	-3.740	17.067	.016
Age	-.098	.081	-1.215	.227	-.258	.062	.015
BZR_entries	.060	.070	.858	.393	-.079	.198	.008
Comorbid_PD	-1.296	1.442	-.899	.371	-4.157	1.566	.008
Type_of_substance_user	.078	.728	.107	.915	-1.368	1.523	.000
Förderschule	.033	1.528	.021	.983	-3.000	3.065	.000
Hauptschule	1.438	.982	1.464	.146	-.511	3.387	.022
Realschule	2.153	1.844	1.167	.246	-1.508	5.813	.014
SUD	-.065	2.515	-.026	.980	-5.055	4.926	.000
AUD	-2.263	2.110	-1.073	.286	-6.450	1.924	.012
Duration	-.004	.005	-.760	.449	-.014	.006	.006

^a^HC3-Methode.

## 4 Discussion

We found that 71 out of 108 patients relapsed within a two-year period, which corresponds to a relapse rate of 65.7%. This locates the results at the upper end of reported recidivism rates across different studies (7). Overall, it can be stated that a large proportion of patients are unable to remain abstinent during forensic mandatory treatment with this study being no exception. The results support Körkel (21), who concluded that recurrence of substance use appears to be a consistent factor during mandatory forensic treatment. At the same time, none of the factors looked at in the regression analysis had an influence on consumption relapses. The sample size fell 10 people short of the recommended minimum to detect medium sized effects. It is possible that the predictors might only have a small effect on relapses and because of the sample size, the model was not able to detect them. If this is the case, the question is just how important these predictors truly are. Future studies should include lager sample sizes, determine the actual effect size of these factors and discuss their relevance.

Concerning the relapse rates, the question arises as to why they are so high, even in highly restrictive settings. One reason is of course the psychopathology of the substance use disorder. A diagnosis of substance use disorder refers to a pathological pattern of cognitive, behavioral and physiological characteristics in which the use of psychotropic substances continues despite considerable long-term disadvantages (29). In addition to a compulsive element in using drugs, an ambivalent treatment motivation and relapses after attempts to quit characterize the disorder (30, 31). Given the nature of substance use disorder, relapses therefore appear to be an inherent part of its psychopathology and by extension its treatment.

While a certain basic risk of relapse is therefore inherent to the disorder, this can be either reduced or increased by internal and external factors, which could explain the differences in individual relapse rates. Each relapse should therefore be considered in the light of the underlying influencing factors and it is important to distinguish, e. g., whether forensic patients relapse due to lack of coping skills or a lack of motivation to remain abstinent (4). Relapses may therefore be seen not primarily as a sign of a lack of motivation but rather as an indicator of an imbalance between individual protective and pathogenic factors. It seems important to analyze the various factors influencing a relapse in order to adapt the treatment to the patient’s needs in the best possible way.

This study was able to show that there are differences in the relapse rate at an individual level. While one third of the patients with recurrence of substance use had only relapsed once, another third relapsed between two and four times and another third more than five times. However, this study cannot provide any reasons as to why certain patients relapsed or why some relapsed more than others. Interestingly, none of the static factors selected predicted relapse which contradicts findings from previous studies. While considering the limitations of the study, static factors alone do not appear to be sufficient to assess the individual risk of relapse. More focus needs to be put on dynamic factors. Schalast (32) found evidence that initial motivation for therapy, high therapy-related trust, compliance and the desire for abstinence are associated with a lower risk of relapse in forensic patients during treatment. A stronger perception of addiction-related impairments by patients in turn leads to a higher risk of relapse. Berthold and Riedemann (1) also found that low treatment motivation negatively affects relapse rates in forensic patients. Although the reviews by Stillman and Sutcliff (33) and Azmi et al. (34) are not from forensic settings, they did show that dynamic factors, such as self-efficacy and support from family and friends, do have an effect on consumption relapse. In another systematic review, adverse emotions, family disputes, a delinquent drug-related environment, social rejection and high availability were reported as reasons for relapse in non-forensic patients with a substance use disorder (35).

According to Scheuschner et al. (18) lack of treatment motivation, lack of familial support and consumption relapse are some of the few predictors of early treatment termination in forensic patients. Given this, it is reasonable to explore if lack of treatment motivation and lack of family support also affect substance use relapse rates during treatment. One study in a forensic setting investigated factors that predict treatment challenging behavior (TCB), which includes disciplinary incidents, escapes and substance relapse during treatment. Overall, younger age, high impulsivity and substitution increase the odds of showing TBC, while a longer prison sentence and monosubstance users lower the risk (36). In this study, however, we were unable to confirm age, number of previous convictions or even the degree of substance use disorder as predictors of substance use. The heterogeneity across different studies in general is already known and can be linked to differences in research methods and construct operationalization (7). Furthermore, a differentiated understanding of substance use during treatment may also be necessary. Substance use is not a uniform phenomenon (10). It has been shown that relapse during forensic therapy does not automatically result in a higher risk of reoffending (22). Future studies on recurrence of substance use during treatment should therefore examine both static and dynamic factors and rethink the conceptualization of substance recurrence during treatment. It could further be investigated whether dynamic and static risk factors can be clustered into profiles, which then allow an assessment of the individual risk of relapsing and possible treatment intervention of the respective patient. For this study, some of the patients who relapsed more than five times might have been less motivated or might suffer from a severe substance use disorder or their needs might not have been properly addressed during treatment. In this context, substance use behavior and its related factors should be further investigated. This includes, for example, the choice of drug. In this study, Cannabinoids, especially Spice and THC, was the most prominent substances consumed across all those who relapsed. This seems to fit well with the generally increased consumption of cannabinoids and that the drug is nowadays more widespread and easier to access than before (37, 38). There is also evidence that cannabis use promotes the use of Spice in non-forensic psychiatric patients (39). Scherbaum et al. (40) report a lifetime prevalence of Spice of 50% in non-forensic patients with a substance use disorder. However, Spice is also highly prevalent in forensic settings. Despite a general downward trend, Spice use in particular remains a major problem in forensic addiction treatment in Germany, as Spice is difficult to detect in tests due to its various chemical structures (41). This might be the reason why forensic inpatients used Spice but it could just as well mean that Spice is cheaper to buy or is easier to smuggle into the clinic. However, this is speculative at this point. On a broader level, the substances consumed and their relevance for reoffending should be considered in particular and further measures should then be taken based on these findings. When facing relapses on an individual level, an adjustment of the therapeutic focus or a reevaluation of the individual relapse prevention strategies should be considered.

Another factor that could influence the heterogeneity of relapse rates might be the clinical setting. It has already been shown that the clinic or the respective court have an influence on treatment outcome (42). When it comes to the clinical setting, there are a number of different factors like different treatment programs or security level across different wards that influence the course of treatment and relapse risk. Thus, such circumstantial factors could also have had an influence on the fluctuations in reported relapse rates across different studies. Clinics with a lower recidivism rate might terminate treatment earlier upon relapsing. However, early treatment termination due to relapses fails to recognize that this poses an additional risk factor for reoffending. Furthermore, relapses are inherent to substance use disorders which are also characterized by its ambivalence towards abstinence. If a patient has a high number of relapses, this could indicate a particularly severe substance use disorder and not necessarily a lack of motivation. Terminating treatment mainly based on relapses should therefore be done cautiously. A close examination of substance use behavior, the consumed substances and its possible relation to the patient’s individual risk of reoffending seems advisable. In this regard, relapses during treatment also offer a good opportunity for therapeutic interventions and the improvement of individual relapse prevention.

Finally, it should be noted that too few studies on the prevalence on recurrence of substance use during treatment have been reported to this date and that further studies are necessary to confirm the findings. This also raises the question as to why there are so few studies on the subject. It is possible that a report on internal hospital prevalence rates is hindered by the assumption that high relapse rates could be interpreted as a result of a failure on the clinic’s end. Future studies should also take a closer look at the prerequisites for mandatory substance use treatment, the substance use behavior of patients at an individual level and the therapeutic services offered in the forensic psychiatric hospitals themselves. There may be a mismatch between what clinics offer and what patients expect from treatment. For example, most forensic patients suffering from addiction do not seek abstinence from every substance or cannot maintain it for long (43). Even after abstinence-oriented forensic treatment, many individuals are unwilling or unable to stay abstinent and continue using multiple substances after release (22). It should not be forgotten that the actual aim of treatment in accordance with § 64 of the German Criminal Code is not to cure the addiction, but to reduce the risk of reoffending. In this context, the cure would merely be a means and not the actual end (43). Maybe the abstinence paradigm is no longer appropriate for some forensic patients, therefore the goal-open treatment approach could be an option after release. Goal-open addiction treatment (*Zieloffene Suchtbehandlung*; ZOS) after release might be one strategy for keeping patients motivated to remain abstinent during forensic treatment. ZOS prioritizes the goals that the patient sets for him- or herself. Seeing addictive behavior as a meaningful attempt to cope with both internal and external stressors in life, respecting the patient’s autonomy (incl. treatment goal and option) and having confidence in the patient to make the best decisions for him- or herself are additional fundamental attitudes that are contributive to ZOS (44). The patient and society itself might benefit from an open-ended addiction treatment approach since only self-desired and pursued goals can lead to positive changes in substance use and the long-term separation of substance usage and reoffending (43).

### 4.1 Limitations

In contrast to existing literature, the regression analysis did not yield any significant results. This was most likely due to a small sample size and a high type-II error. Therefore our results do not question the validity of the investigated predictors. Future studies should therefore include larger sample sizes. Relapses were identified using standard positive laboratory drug tests, drug test refusal or a patients’ own admission of consuming drugs. Therefore the data was free from personal bias such as any clinicians’ mere observations of patient behavior. Tests are either scheduled by the staff or carried out whenever there is sufficient indication of intoxication. Still, the reported number of relapses can only be an approximation to the true relapse rates. It cannot be ruled out that patients may relapse without being noticed. The accuracy of the reported relapse rates is further reduced by the irregular testing within the clinic and/or between its respective wards. For instance, some wards might test the patients more often than others and are thereby more likely to detect relapses more often. The reported relapse rates are therefore only an approximation of the true value. **Table 4** shows that tests were carried out with varying frequency over the different years and it is therefore possible that some relapses remained undetected in a year with very fewer tests. To count a drug test refusal as a relapse should also be viewed critically since it harbors the risk that there are patients who have not actually relapsed at all and refused the test for a different reason. However, since the data contained only patients who already left the clinic, it was not possible to inquire as to their reasons to refuse a drug test some time ago. Finally, it should be noted that the treatment concept of the respective clinic also influences the reported relapse rate. For instance, in the Clinic for Forensic Psychiatry Rostock, patients are not discontinued after a set number of relapses which leaves room for more relapses over the course of treatment.

### 4.2 Conclusion

This study investigated the substance use relapse rates among forensic inpatients in one hospital in Germany, the specific substances taken during relapses and the factors that affect the relapse rate. A high relapse rate is a reality of forensic addiction treatment. Ultimately, substance use relapses are intrinsic to the psychopathology of substance use disorder. Relapses should be approached constructively during treatment, as they should be seen as an indicator of an imbalance of a patients’ protective and pathogenic factors. The number and frequency of relapses should therefore always be evaluated with regard to the existing protective factors and risk factors of each individual. Static predictors alone are not sufficient to fully explain substance relapse, might only have a small effect on relapsing and are unfit to constitute a basic risk level alone. It is therefore necessary to take a closer look at dynamic factors that affect recurrence of substance use. Considering the findings of this research, future studies should investigate the patient’s substance use behavior during treatment (e.g. reason for relapsing or choice of drug), identify and investigate the relationship between individual and context factors and how they affect relapses and uncover possible treatment interventions that might reduce relapse rate, dropout rate and criminal recidivism.

The evidence on the underlying the causes of recurrence of substance use is to this day not conclusive, even though research on this subject is being done for decades (27). One reason for this heterogeneity is that the variables aren’t operationalized in the same way across different studies (7). It is therefore imperative to focus on standardizing the operationalization of relapse, consumption behavior and its predictors before further quantitative studies are conducted. Furthermore it is important to ensure that the sample size is sufficiently large, particularly in light of the results of this study.

## Data Availability

The original contributions presented in the study are included in the article/**Supplementary Material**. Further inquiries can be directed to the corresponding author.
