# Supplementary material for: Prevalence of alcohol and substance use relapse in forensic inpatients with substance use disorders in Germany
Source: Front Psychiatry. 2025 Oct 17;16:1663413. doi: 10.3389/fpsyt.2025.1663413 (PMC12575263; doi:10.3389/fpsyt.2025.1663413)
Supplement: Supplementary file 1 [file DataSheet1.pdf]

Frontiers Interactive Review – Supplementary material for the Revision of *„Prevalence of Alcohol and Substance Use Relapse in Forensic Inpatients with Substance Use Disorders in Germany"*

Dear Sir or Madam,

please find below the supplementary material for the multiple regression analysis.

**Methods – Multiple Regression analysis**

## 1. A priori – Power analysis

Small effect

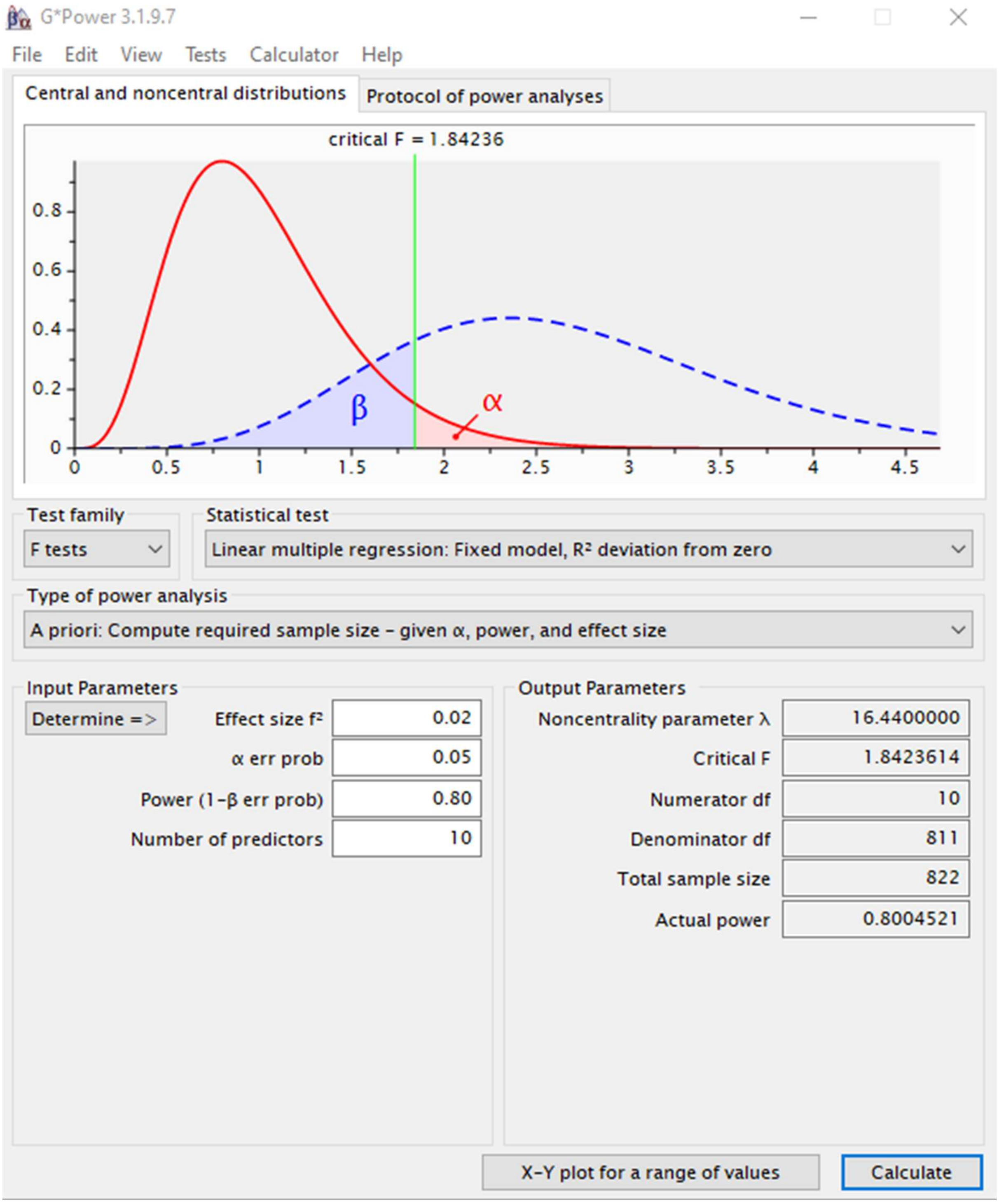

Medium effect

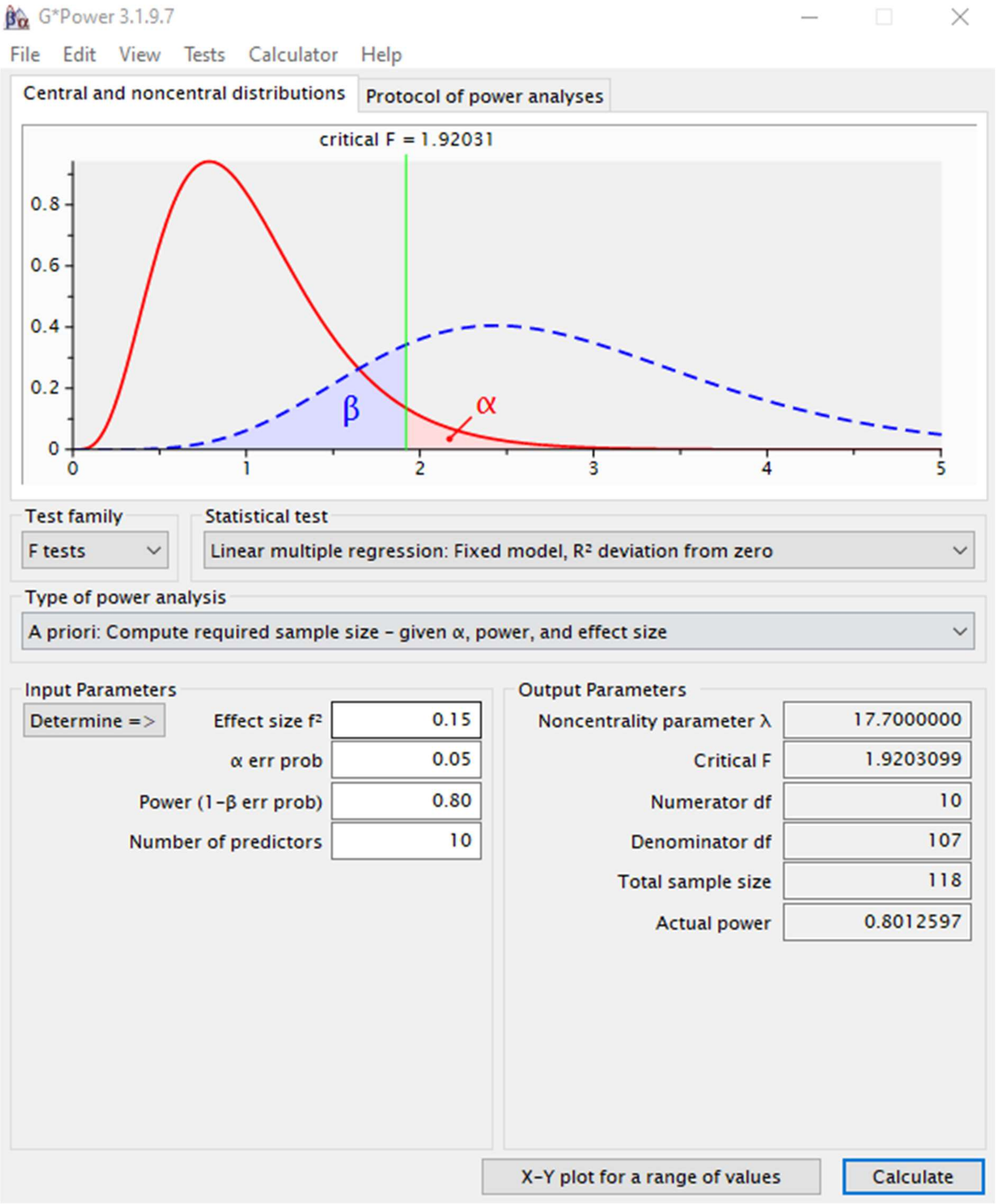


**Post hoc – Power analysis**

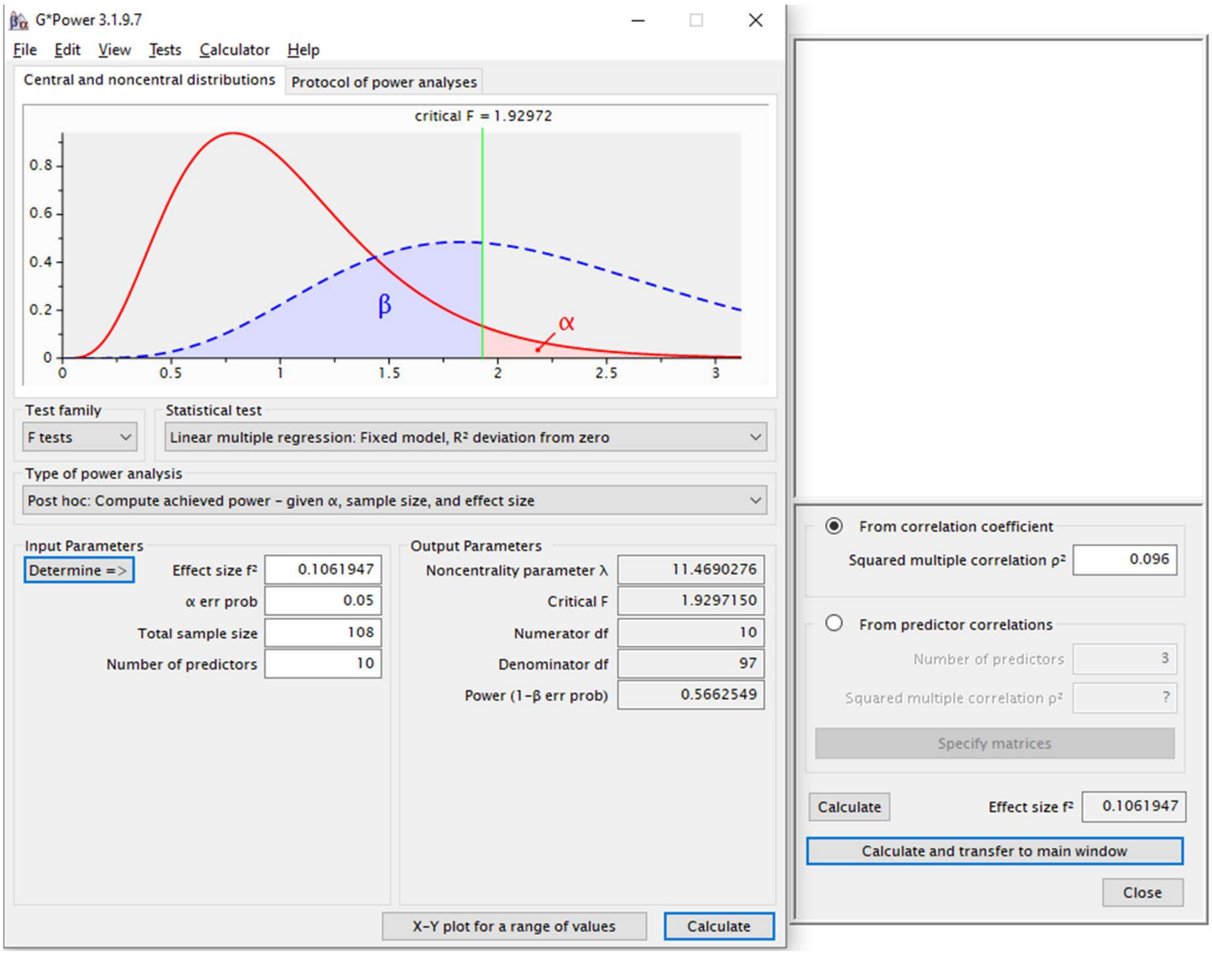


Power is at ~ 56.6% which leaves a high Type II (~43.4%) Error. Chances are high that the model was just not able to detect the effect. You should have a Power of at least 80% (Döring, 2023).

## Assumptions

### I. Linear relationship (metric variables only)

According to the scatterplots, the linear relationship for the metric independent variables can be assumed. Linear relationship for dummy-codes variables was not tested since they are not metric.

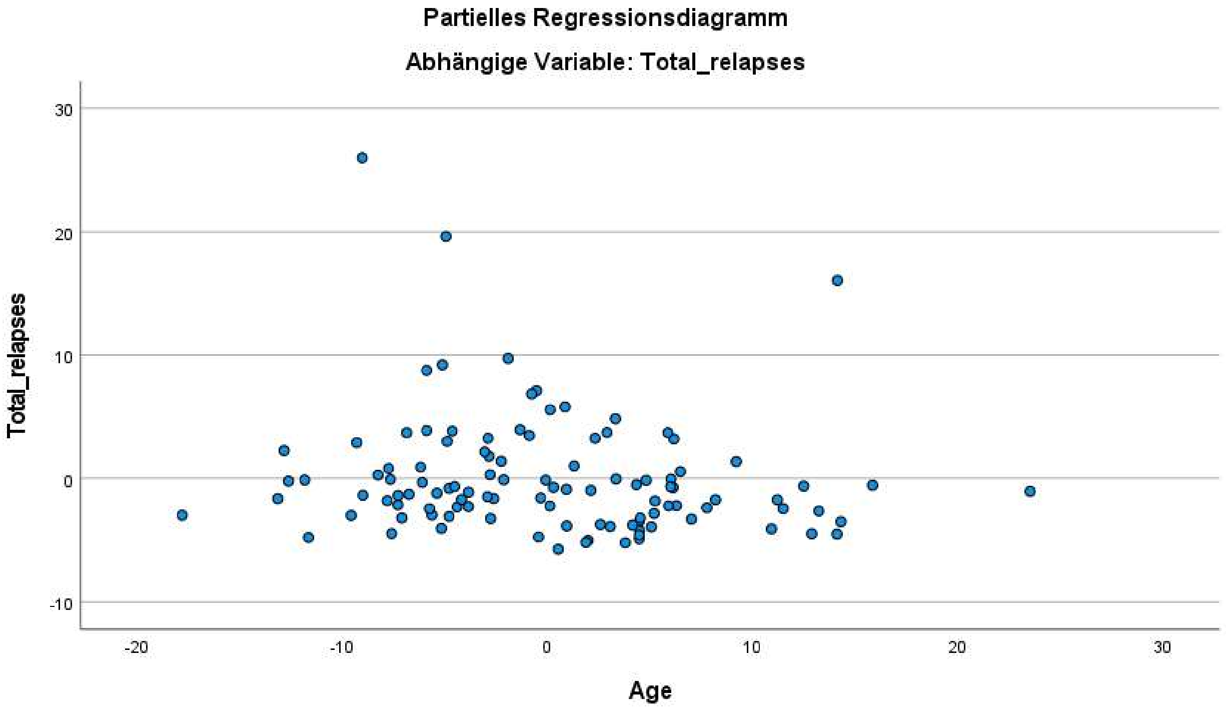
Partielles Regressionsdiagramm
Abhängige Variable: Total_relapses
Total_relapses
30
20
10
0
-10
-20
-10
0
10
20
30
Age

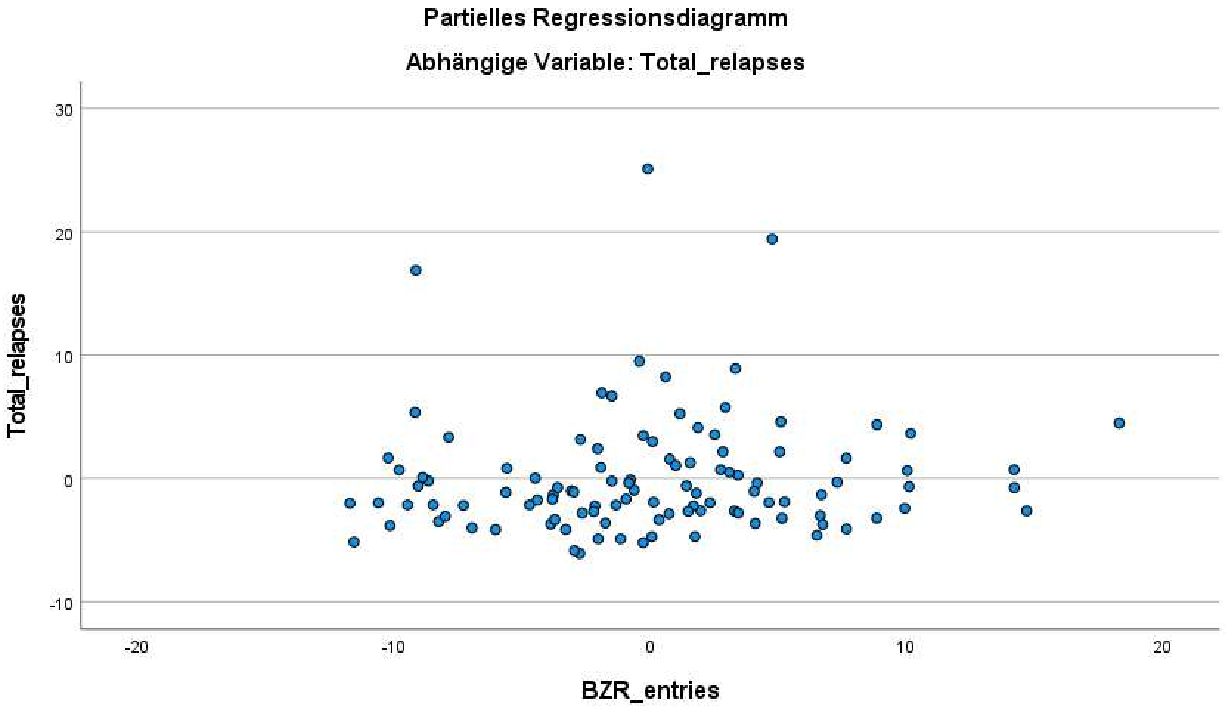
Partielles Regressionsdiagramm
Abhängige Variable: Total_relapses
Total_relapses
30
20
10
0
-10
-20
-10
0
10
20
BZR_entries

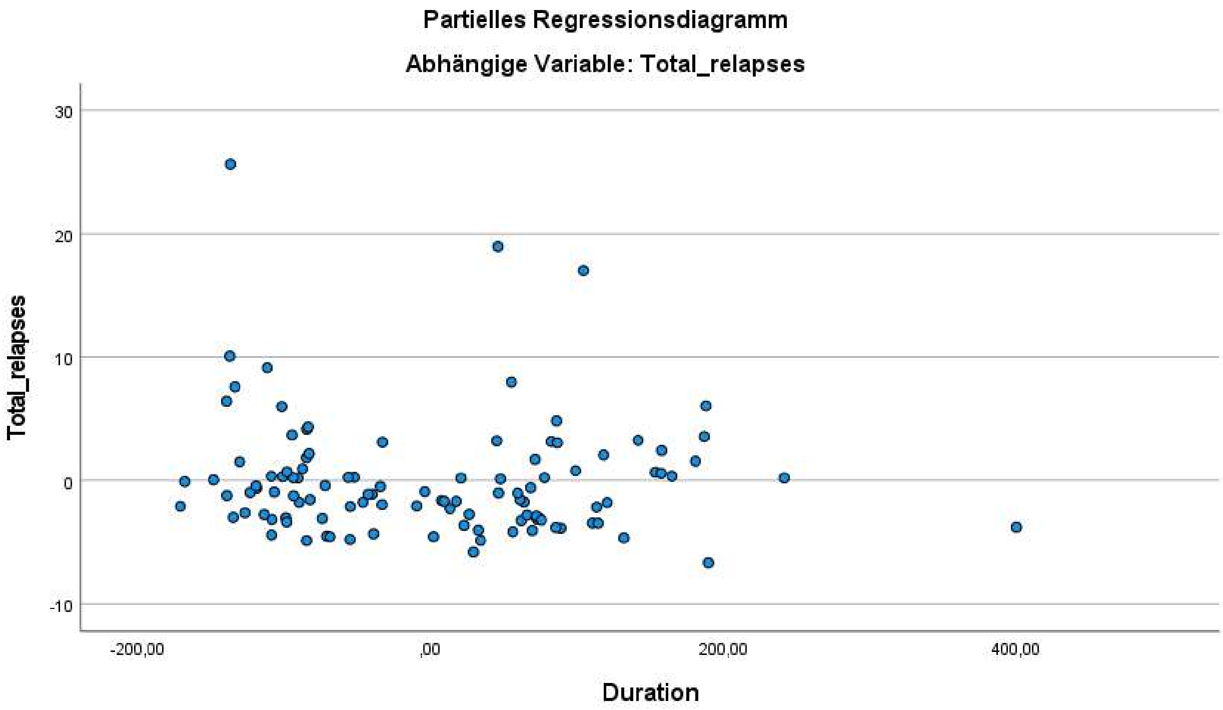


## II. Independence of variables

## III. Normality

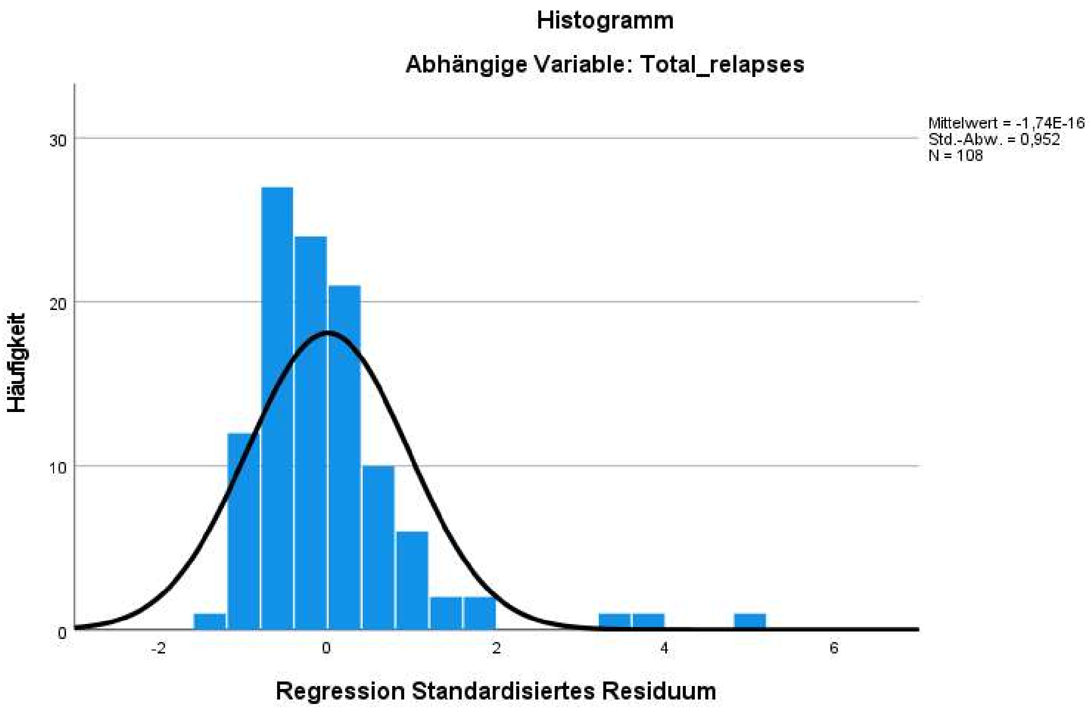

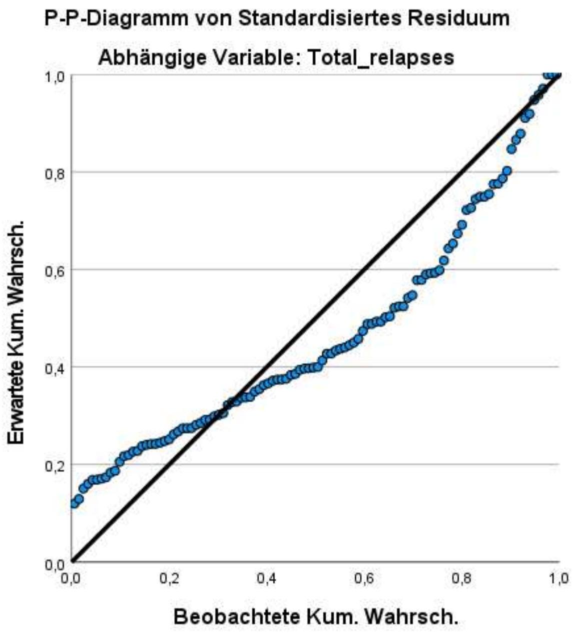


P-P-Plot shows assumption is not violated since the residuals fit along the line nicely.

## IV. Independence of errors

Assumption is met because Durbin-Watson value is between 1 and 3.

| Model | R | R Square | Adjusted R Square | Standard Error of the Estimate | Durbin-Watson-Statistic |
|---|---|---|---|---|---|
| 1 | ,310[a] | ,096 | ,003 | 5,044 | 1,983 |

## V. Homoscedasticity

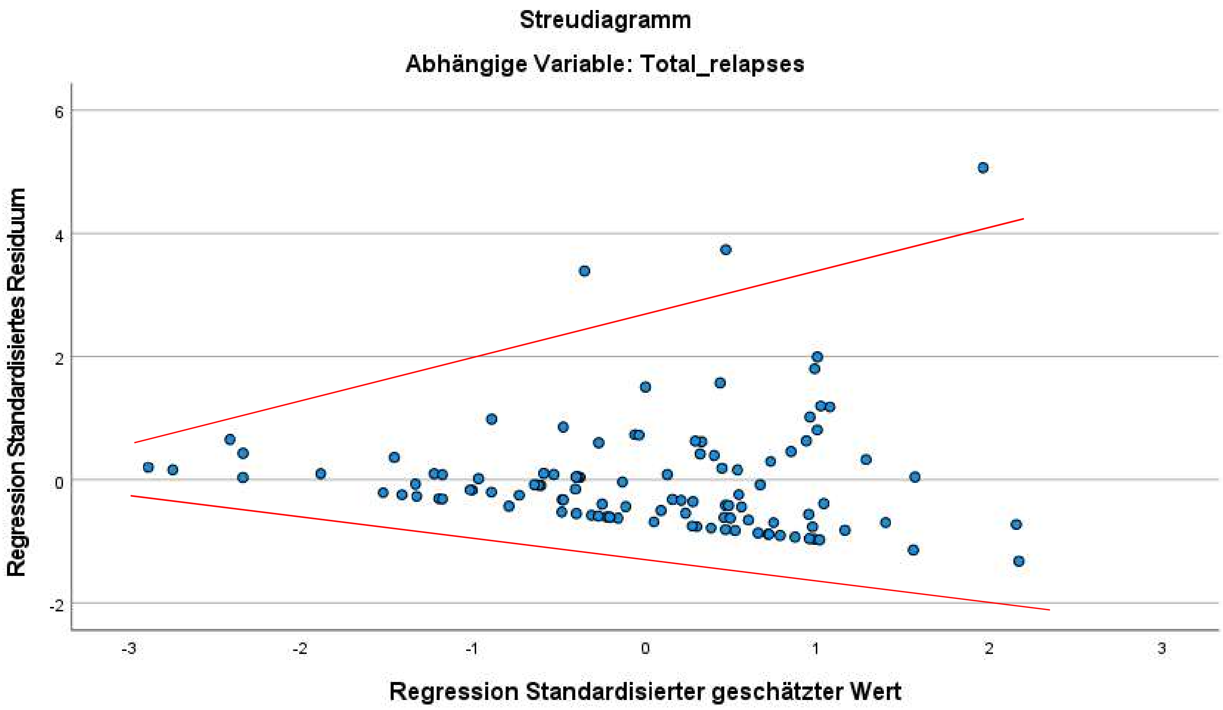


Assumption might be violated. Therefore we used the HC3-Method to control for heteroscedasticity afterwards.

## VI. Multicollinearity

The collinearity statistics, Tolerance and VIF (Variance Inflation Factor) check the assumption of multicollinearity. As a rule of thumb, if VIF <10 and tolerance is >0.1 then assumption is not violated.

**Coefficients[a]**

| Modell | | non standarized Coefficients | | Standardized Coefficients | | | 95,0% Confidence Interval for B | | Colinerarity Statistics | |
|---|---|---|---|---|---|---|---|---|---|---|
| | | Regression Coefficients (B) | Standard Error | Beta | t | Sig. | Lower Bound | Upper Bound | Tolerance | VIF |
| 1 | (Konstante) | 6,664 | 2,874 | | 2,318 | ,023 | ,959 | 12,369 | | |
| | Age | -,098 | ,067 | -,167 | -1,468 | ,145 | -,230 | ,034 | ,723 | 1,383 |
| | BZR_entries | ,060 | ,079 | ,082 | ,755 | ,452 | -,097 | ,217 | ,798 | 1,254 |

| | | | | | | | | | |
|---|---|---|---|---|---|---|---|---|---|
| | Comorbid_PD | -1,296 | 1,139 | -,118 | -1,138 | ,258 | -3,556 | ,964 | ,872 | 1,147 |
| | Type_of_substance_user | ,078 | ,932 | ,009 | ,084 | ,933 | -1,771 | 1,927 | ,733 | 1,364 |
| | Förderschule | ,033 | 2,055 | ,002 | ,016 | ,987 | -4,047 | 4,112 | ,813 | 1,230 |
| | Hauptschule | 1,438 | 1,214 | ,141 | 1,184 | ,239 | -,972 | 3,848 | ,653 | 1,531 |
| | Realschule | 2,153 | 1,443 | ,175 | 1,491 | ,139 | -,712 | 5,017 | ,675 | 1,482 |
| | SUD | -,065 | 1,498 | -,005 | -,043 | ,966 | -3,037 | 2,908 | ,574 | 1,741 |
| | AUD | -2,263 | 2,197 | -,141 | -1,030 | ,305 | -6,623 | 2,097 | ,494 | 2,024 |
| | Duration | -,004 | ,005 | -,086 | -,859 | ,393 | -,013 | ,005 | ,931 | 1,074 |

a. Abhängige Variable: Total_relapses

## Results

We conducted a multiple regression analysis which yielded no significant results in the F-Test (p>.05).

**ANOVA[a]**

| Model | | Sum of Squares | df | Mean Square | F | Sig. |
|---|---|---|---|---|---|---|
| 1 | Regression | 262,093 | 10 | 26,209 | 1,030 | ,424[b] |
| | Residual | 2467,574 | 97 | 25,439 | | |
| | Total | 2729,667 | 107 | | | |

a. Dependant Variable: Total_relapses

b. Predictors: (Constant), Duration, BZR_entries, SUD, Hauptschule, Comorbid_PD, Förderschule, Type_of_substance_user, Age, Realschule, AUD

Adjusted R square show negligibly small effect and shows that the model (if significant) can explain 0.3% of the Variance of the dependent variable.

**Model Summary**

| Model | R | R Square | Adjusted R Square | Standard Error of the Estimate | Durbin-Watson-Statistic |
|---|---|---|---|---|---|
| 1 | ,310a | ,096 | ,003 | 5,044 | 1,983 |

Frontiers Interactive Review – Supplementary material for the Revision of *„Prevalence of Alcohol and Substance Use Relapse in Forensic Inpatients with Substance Use Disorders in Germany“*

We used HC3-Method to control for heteroscedasticity:

**Parameter estimates with robust standard errors**

Dependent Variable: Total_relapses

| Parameter | Regression Coefficient (B) | Robuster Standard Error[a] | t | Sig. | 95% Confidence Interval Lower Bound | 95% Confidence Interval Upper Bound | Partial Eta Squared |
|---|---|---|---|---|---|---|---|
| Constant | 6,664 | 5,242 | 1,271 | ,207 | -3,740 | 17,067 | ,016 |
| Age | -,098 | ,081 | -1,215 | ,227 | -,258 | ,062 | ,015 |
| BZR_entries | ,060 | ,070 | ,858 | ,393 | -,079 | ,198 | ,008 |
| Comorbid_PD | -1,296 | 1,442 | -,899 | ,371 | -4,157 | 1,566 | ,008 |
| Type_of_substance_user | ,078 | ,728 | ,107 | ,915 | -1,368 | 1,523 | ,000 |
| Förderschule | ,033 | 1,528 | ,021 | ,983 | -3,000 | 3,065 | ,000 |
| Hauptschule | 1,438 | ,982 | 1,464 | ,146 | -,511 | 3,387 | ,022 |
| Realschule | 2,153 | 1,844 | 1,167 | ,246 | -1,508 | 5,813 | ,014 |
| SUD | -,065 | 2,515 | -,026 | ,980 | -5,055 | 4,926 | ,000 |
| AUD | -2,263 | 2,110 | -1,073 | ,286 | -6,450 | 1,924 | ,012 |
| Duration | -,004 | ,005 | -,760 | ,449 | -,014 | ,006 | ,006 |

a. HC3-Methode

Non-significant result since p>.05 and confidence intervals include 0.
